# A Stepwise NaHSO_3_ Addition Mode Greatly Improves H_2_ Photoproduction in *Chlamydomonas reinhardtii*

**DOI:** 10.3389/fpls.2018.01532

**Published:** 2018-10-31

**Authors:** Lanzhen Wei, Xin Li, Baoqiang Fan, Zhaoxing Ran, Weimin Ma

**Affiliations:** College of Life and Environment Science, Shanghai Normal University, Shanghai, China

**Keywords:** NaHSO_3_, stepwise mode, anaerobic environment, PSII activity, H_2_ photoproduction, *Chlamydomonas reinhardtii*

## Abstract

NaHSO_3_ addition greatly increases the yield of H_2_ photoproduction in a unicellular green alga *Chlamydomonas reinhardtii* through removing O_2_ and activating hydrogenase but significantly impairs the activity of PSII, an electron source for H_2_ photoproduction. Here, a stepwise addition mode of total 13 mM NaHSO_3_, an optimal concentration for H_2_ photoproduction of *C. reinhardtii* identified in a previous one step addition method, significantly improved H_2_ photoproduction. Such improvement was believed to be the result of increased residual PSII activity in an anaerobic background, but was at least independent of two alternative electron sinks for H_2_ photoproduction, cyclic electron transport around PSI and CO_2_ assimilation. Based on the above results, we propose that increased residual PSII activity in an anaerobic environment is an efficient strategy to enhance H_2_ photoproduction in *C. reinhardtii*, and the stepwise NaHSO_3_ addition mode is a case study in the strategy.

## Introduction

With the increasing awareness of fossil fuel depletion and global warming, efforts have been undertaken to develop clean and sustainable energy sources (McKendry, 2002). Molecular hydrogen (H_2_) is one of the potential future energy sources (Hansel and Lindblad, 1998; Momirlan and Veziroglu, 2002). *C. reinhardtii*, a unicellular green alga, has been recognized as an ideal system for sustainable H_2_ photoproduction under anaerobic conditions; however, this alga cannot efficiently and continuously produce H_2_ in an aerobic environment because its H_2_ase is extremely sensitive to O_2_ (Ghirardi et al., 1997). To activate H_2_ase for sustainable and efficient H_2_ photoproduction in *C. reinhardtii*, therefore, numerous strategies have been extensively developed mainly through engineering O_2_ tolerance in H_2_ase (Flynn et al., 2002; Liebgott et al., 2011; Wu et al., 2011) or decreasing O_2_ content around H_2_ase (Melis et al., 2000; Kruse et al., 2005; Surzycki et al., 2007; Wu et al., 2010; Xu et al., 2011; Jurado-Oller et al., 2015; Xiong et al., 2015; Shu et al., 2018). Meanwhile, our studies demonstrate that NaHSO_3_ addition strategy is capable of decreased the O_2_ content around H_2_ase, thereby activating the enzyme activity and promoting H_2_ photoproduction (Wang et al., 2010; Ma et al., 2011; Wei et al., 2017). This strategy can result in an approximately 10-fold or 200-fold increase in H_2_ photoproduction in the nitrogen-fixing cyanobacterium *Anabaena* sp. strain PCC 7120 (Wang et al., 2010) or the unicellular green alga *C. reinhardtii* (Ma et al., 2011; Wei et al., 2017), respectively. Despite these increases, this yield is still not sufficient to meet the requirements of industrial applications. Thus, extensive optimization of this NaHSO_3_ addition strategy is necessary to increase H_2_ photoproduction in *C. reinhardtii* further.

The yield of H_2_ photoproduction caused by sulfur deprivation is also not sufficient to meet the requirements of industrial applications. Under sulfur deprivation conditions, therefore, many strategies have been developed to improve the yield of H_2_ photoproduction in *C. reinhardtii* via metabolic and genetic engineering (for review, see Esquível et al., 2011; Dubini and Ghirardi, 2015). Among them, increased residual photosystem II (PSII) activity was found to play a vital role in efficient H_2_ photoproduction (Zhang et al., 2002; Kosourov et al., 2005; Kim et al., 2010; Volgusheva et al., 2013; Grewe et al., 2014; Steinbeck et al., 2015; Chen et al., 2016), since the PSII activity is significantly impaired by sulfur deprivation (Melis et al., 2000). Similarly, in the NaHSO_3_ addition background, the PSII activity is also significantly impaired (Wang et al., 2010). To test whether increased residual PSII activity in the NaHSO_3_ addition background can also enhance H_2_ photoproduction, we monitored the accumulated H_2_ level and residual PSII activity in the stepwise mode of total 13 mM NaHSO_3_, an optimal concentration for H_2_ production of *C. reinhardtii* identified in a previous one step addition method (Ma et al., 2011). We also measured the content of dissolved O_2_ and activities of two alternative electron sinks for H_2_ photoproduction, CET and CO_2_ assimilation, in the stepwise NaHSO_3_ addition mode. Our results demonstrate that the stepwise NaHSO_3_ addition mode evidently enhances the yield of H_2_ photoproduction in *C. reinhardtii*; such enhancement is mostly the result of increased residual PSII activity in an anaerobic environment, but is at least independent of two alternative electron sinks for H_2_ photoproduction.

## Materials and Methods

### Culture Conditions

Cells of *C. reinhardtii* (CC-503 strain) were cultured at 25°C in TAP medium (Harris, 1989). The medium was buffered with Tris–HCl (20 mM; pH 7.3), bubbled with air under continuous illumination with cool-white fluorescent lamps (40 μmol photons m^-2^s^-1^), and inoculated with approximately 8.1 × 10^4^ cells mL^-1^ of *C. reinhardtii* (inoculum size, 1%).

### Sample Preparation and NaHSO_3_ Addition

**FIGURE 1:**
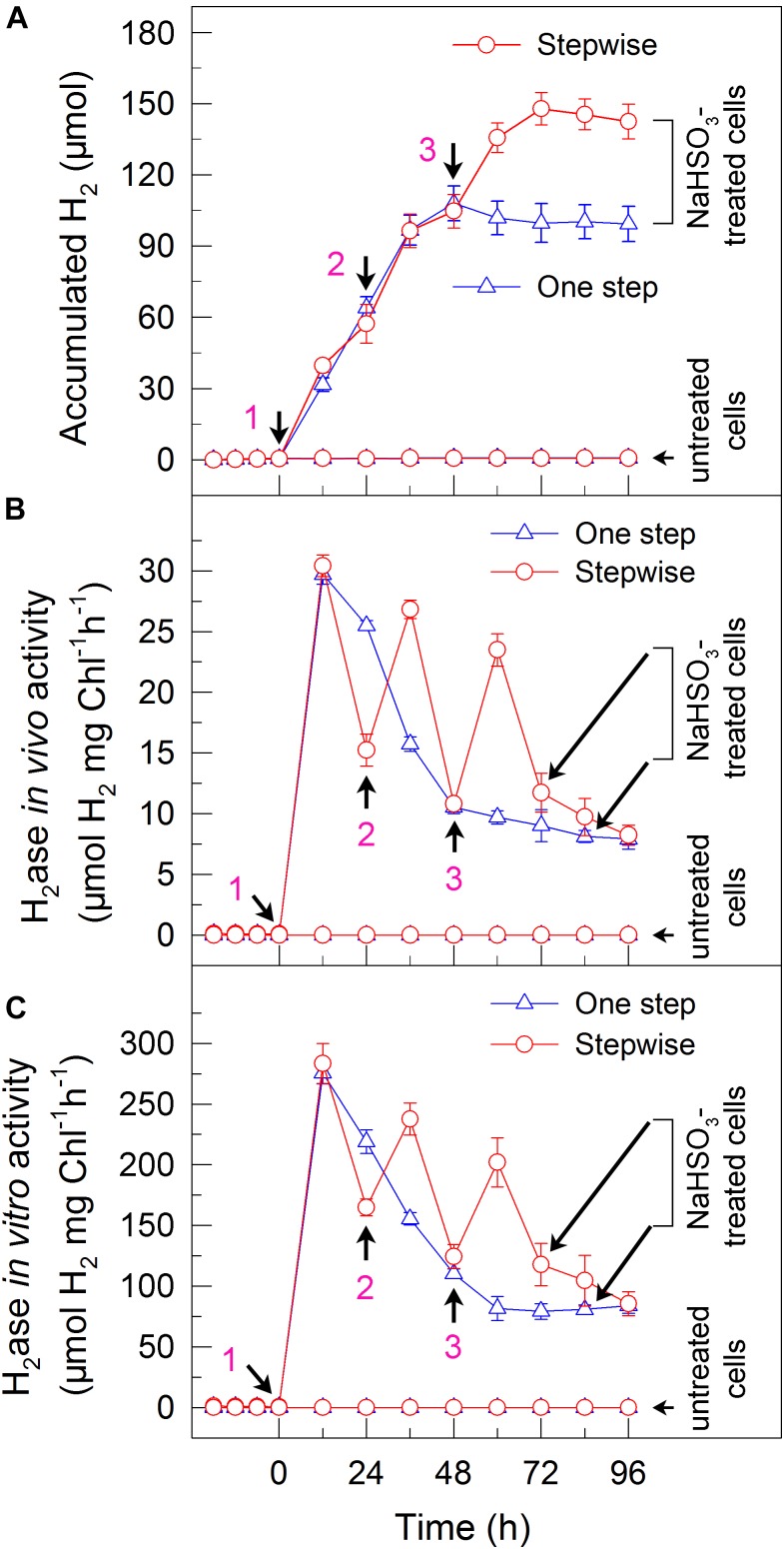
A stepwise NaHSO_3_ addition mode significantly increases **(A)** H_2_ photoproduction, and **(B)** *in vivo* and **(C)** *in vitro* H_2_ase activity in *C. reinhardtii*. After cells were statically pre-cultured under continuous illumination of 200 μmol photons m^-2^s^-1^ for 36 h, total 13 mM of NaHSO_3_ was directly or step by step added to the serum bottles, as indicated by arrows 1–3 and described in Table 1. Values are means ± SD (*n* = 5).

**FIGURE 2:**
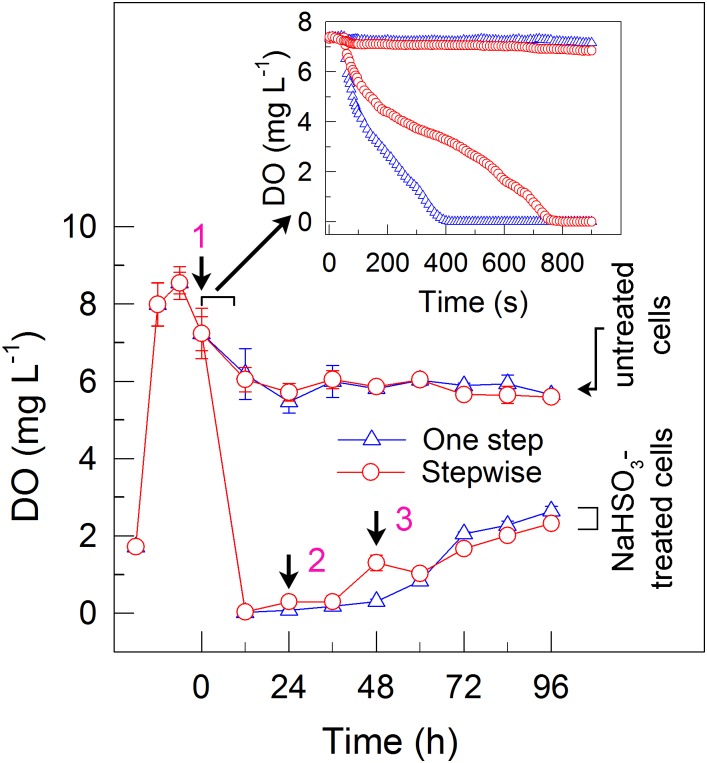
A stepwise NaHSO_3_ addition mode influences the content of dissolved oxygen (DO) in *C. reinhardtii*. Pre-culture conditions of cells and addition modes of NaHSO_3_ are shown in the legend of Figure 1. Values are means ± SD (*n* = 5).

Cells of *C. reinhardtii* were continuously illuminated by growth light of 40 μmol photons m^-2^s^-1^ and were cultured in 0.5 L of TAP medium for 2 days with bubble aeration (*A*_750_ = 0.8–1.0), after which a fixed volume of cells containing 300 μg of Chl was transferred to 60 mL serum bottles (30 mL head space and 30 mL cells) with rubber seals. After cells were statically pre-cultured under continuous illumination of 200 μmol photons m^-2^s^-1^ for 36 h, total 13 mM of NaHSO_3_ was directly or step by step added to the serum bottles, as indicated in Figures 1, 2, Supplementary Figure S1, and described in Table 1, with Lin of 5 mM (final concentration) or AA of 10 μM (final concentration) or GA of 2 mM (final concentration) or DCMU of 20 μM (final concentration) or not. Subsequently, the cells were still illuminated at 200 μmol photons m^-2^s^-1^ or were incubated in the dark to induce the production of H_2_.

**Table 1:** A table schematically represents one step method and stepwise mode of total 13 mM NaHSO_3_.

NaHSO_3_ addition	NaHSO_3_ (mM)			
	**Arrow 1**	**Arrow 2**	**Arrow 3**	**Total**

One step method	13	0	0	13
Stepwise mode	9	2	2	13

**FIGURE 3:**
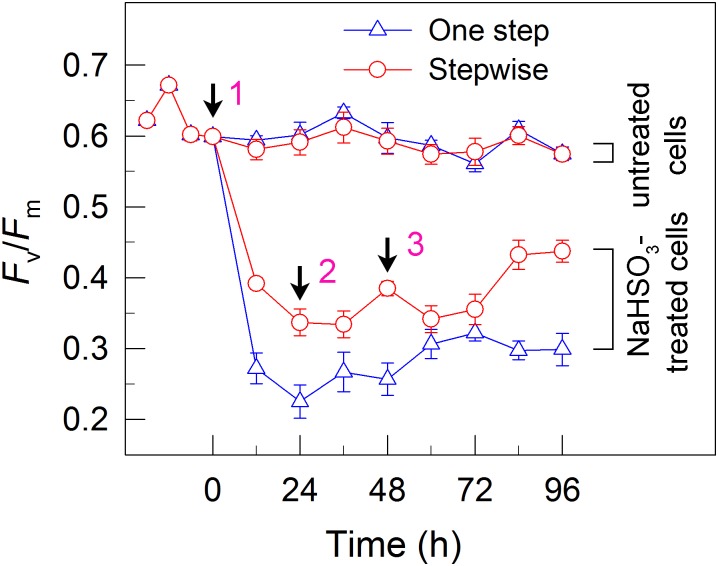
A stepwise addition mode alleviates the inhibitory effects of NaHSO_3_ on PSII activity in *C. reinhardtii*. The Chl concentration was adjusted to 10 μg mL^-1^ before measurement. PSII activity was evaluated by the *F*_v_/*F*_m_ values. Values are means ± SD (*n* = 5).

**FIGURE 4:**
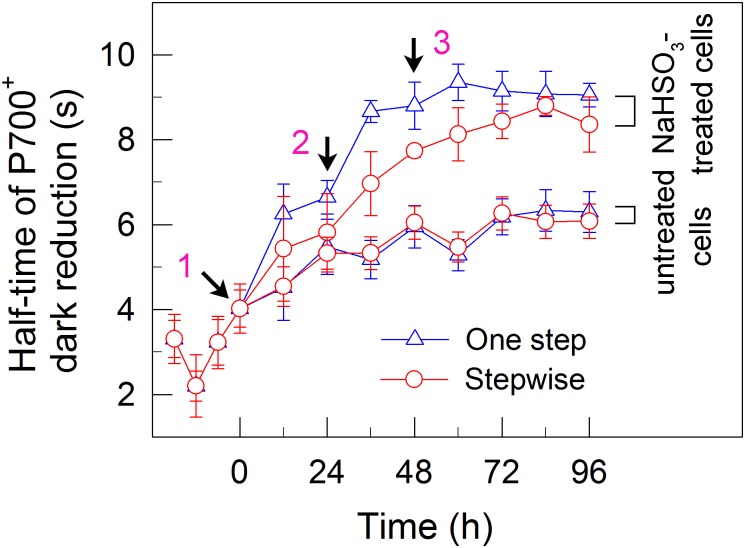
A stepwise addition mode slightly alleviates the inhibitory effects of NaHSO_3_ on cyclic electron transport around PSI in *C. reinhardtii*. The rate of cyclic electron transport around PSI was judged by half-time of P700^+^ dark reduction. Values are means ± SD (*n* = 5).

**FIGURE 5:**
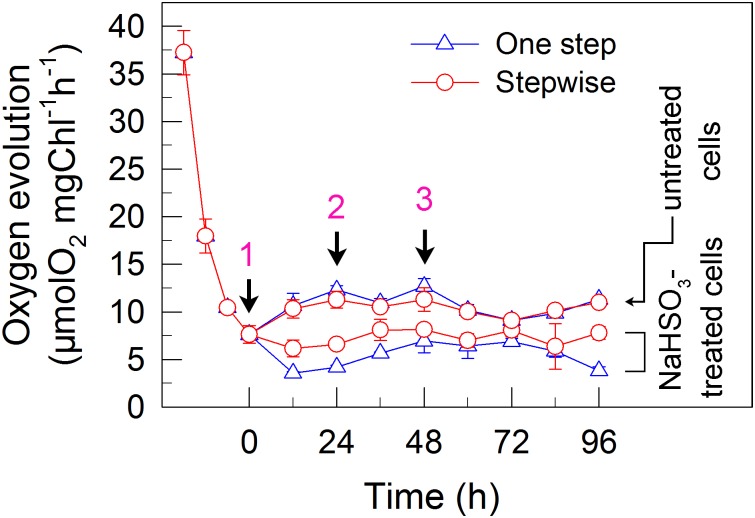
A stepwise addition mode slightly alleviates the inhibitory effects of NaHSO_3_ on CO_2_ assimilation in *C. reinhardtii*. Activity of CO_2_ assimilation was assessed by photosynthetic production of O_2_ with NaHCO_3_ as an artificial electron acceptor. Values are means ± SD (*n* = 5).

*Arrows 1–3 shown in Figures 1–5 and both arrows have a 24 h interval.*

### Monitoring H_2_ Photoproduction

At predetermined time intervals, 200 μL of gas samples were withdrawn from the bottles using a gas-tight syringe and injected into a gas chromatograph (Agilent 7890A; Agilent Technologies Inc., United States) with a thermal conductivity detector for determining the concentrations of H_2_, O_2_, and N_2_ simultaneously. The column of the gas chromatograph was a molecular sieve column (type 5Å; 2 m × 1/8 mm), and argon was used as the carrier gas.

### H_2_ase Activity Assay

*In vivo* and *in vitro* H_2_ase activity was monitored as described earlier (Ma et al., 2011; Wei et al., 2013, 2017) with some modifications. In brief, 1 mL cell suspension samples upon exposure to 200 μmol photons m^-2^s^-1^ were anaerobically withdrawn from the 60 mL serum bottles at designated time points (see Figures 1B,C) and then injected into 10 mL glass vials. To measure *in vivo* H_2_ase activity, the cell suspension samples were immediately purged with argon gas for 1 min to eliminate the inhibitory effect of O_2_ on the H_2_ase activity. The cell suspension samples were then placed in a 25°C water bath for 1 h and shaken continuously (150 rpm) whilst exposed to a constant light of 200 μmol photons m^-2^s^-1^. To measure *in vitro* H_2_ase activity, we used vials containing 1 mL of 10 mM oxidized MV prepared in O_2_-free 50 mM Tris buffer (for pH 7.1–9.0) and 0.2% (*w*/*v*) Triton X-100. The reaction was started when MV was reduced by the addition of 100 μL of 100 mM anaerobic sodium dithionite in 0.03 N NaOH. This assay was performed at 37°C in the dark for 20 min. We determined the amount of H_2_ produced in the headspace of the glass vial by gas chromatography, and the rate of H_2_ production was calculated on the basis of the total Chl content in the glass vial, unless otherwise indicated.

### Dissolved Oxygen Measurement

Dissolved oxygen was monitored as described earlier (Wei et al., 2017). In brief, a DO meter (Orion Star A213, Thermo Scientific, Untied States) was used to monitor the DO attenuation process after the addition of NaHSO_3_ to the cell suspension cultures of *C. reinhardtii*. The DO meter was corrected before each measurement. The DO meter probe was placed in the middle of the cell suspension cultures and the data were recorded at several designated time points.

### Chl Fluorescence and P700 Analysis

The yields of Chl fluorescence at a steady-state of electron transport were measured at room temperature using a Dual-PAM-100 monitoring system (Walz, Effeltrich, Germany) equipped with an ED-101US/MD unit (Schreiber et al., 1986; Ma et al., 2008; Wei et al., 2013, 2017). Minimal fluorescence at open PSII centers in the dark-adapted state (*F*_o_) was excited by a weak measuring light (650 nm) at a photon flux density of 0.05–0.15 μmol photons m^-2^s^-1^. A saturating pulse of red light (600 ms, 10,000 μmol photons m^-2^s^-1^) was applied to determine the maximal fluorescence at closed PSII centers in the dark-adapted state (*F*_m_). *F*_v_/*F*_m_ was evaluated as (*F*_m_-*F*_o_)/*F*_m_ (Kitajima and Butler, 1975; Wei et al., 2013, 2017).

The reduction of P700^+^ in darkness was measured with the aforementioned Dual-PAM-100 fluorometer by monitoring absorbance changes at 830 nm and using 875 nm as a reference. Cells were kept in the dark for 2 min, and 10 μM of DCMU was added to the cell suspension cultures prior to the measurement. The P700 was oxidized by far-red light with a maximum at 720 nm from a light-emitting diode lamp for 30 s, and the subsequent re-reduction of P700^+^ in the dark was monitored and its half-time was calculated.

### Oxygen Evolution Activity

Oxygen production in intact *C. reinhardtii* cells by photosynthesis was determined at 25°C by monitoring the changes in O_2_ levels with a Clark-type oxygen electrode (Hansatech Instruments, King’s Lynn, United Kingdom). Prior to the measurements, 10 mM of NaHCO_3_ was added to the cell suspension cultures. The intensity of light used for the measurements was 1,000 μmol photons m^-2^s^-1^.

## Results

### A Stepwise NaHSO_3_ Addition Mode Significantly Increases the Yield of H_2_ Photoproduction in *C. reinhardtii*

To test whether a stepwise NaHSO_3_ addition mode can enhance the yield of H_2_ photoproduction, we monitored accumulated H_2_ amounts in the one step addition method and stepwise mode of total 13 mM NaHSO_3_ (hereafter one step method and stepwise mode, respectively; see Table 1), an optimal concentration for H_2_ photoproduction of *C. reinhardtii* identified in a previous one step method (Ma et al., 2011). Compared to the one step method, stepwise mode evidently enhanced the yield of H_2_ photoproduction (Figure 1A and Table 2). The H_2_ level in stepwise mode was approximately 1.5 times greater than that in one step method and, approximately 350 times greater than that in untreated cells (Figure 1A and Table 2). This was confirmed by the results of *in vivo* (Figure 1B) and *in vitro* (Figure 1C) H_2_ase activity. We therefore conclude that the stepwise mode considerably improves H_2_ photoproduction in the green alga *C. reinhardtii*.

**Table 2:** Comparison of H_2_ photoproduction characteristics in *C. reinhardtii* between one step method and stepwise mode.

NaHSO_3_ addition	Vmax (μmol H_2_ mg Chl^-1^h^-1^)^1^	Amax (relative; %)^2^	Time (day)^3^
One step method	11.8 ± 0.7	100 ± 6.9	50.2 ± 3.2
Stepwise mode	14.7 ± 1.2	145.6 ± 5.1	76.1 ± 2.8

*^1^Vmax indicates the maximum velocity of H_2_ photoproduction. ^2^Amax indicates the maximum accumulated H_2_ (calculated from Figure 1A) and its value in one step method was taken as 100%. ^3^Time represents the time of continuous H_2_ production, which was calculated from Figure 1A.*

### The Stepwise Mode Can Also Establish an Anaerobic Environment

To elucidate the mechanism underlying the increase in the H_2_ yield under stepwise mode, we monitored the dissolved O_2_ (DO) content in the cell suspension cultures of *C. reinhardtii*. The results indicated that addition of total 13 mM NaHSO_3_ to the cell suspension cultures in both the one step method and stepwise mode can similarly create an anaerobic environment (Figure 2), although the stepwise mode was slightly slow to generate an anaerobic environment when compared to the one step method (insert in Figure 2). It is worthy of note that, when an initial concentration of NaHSO_3_ in stepwise mode was less than or equal to 7 mM, the cell suspension cultures did not enter or maintain an anaerobic environment, which evidently suppressed the increase of H_2_ photoproduction in the stepwise mode (data not shown). Based on the above results, we propose that the stepwise mode is necessary to operate in an anaerobic environment as an efficient strategy for H_2_ photoproduction in *C. reinhardtii*.

### The Stepwise Mode Maintains a Relatively High Residual Activity of Electron Source for H_2_ Photoproduction

To understand why the stepwise mode can increase the yield of H_2_ photoproduction, we measured the activity of PSII, an electron source for H_2_ photoproduction. After the addition of total 13 mM NaHSO_3_ to the cell suspension cultures, the residual activity of PSII was much higher in stepwise mode than that in one step method, as evaluated by the *F*_v_/*F*_m_ values (Figure 3). This was supported by the results that the stepwise mode maintained a slightly high DO content at an efficient H_2_ production stage when compared to the one step method (Figure 2). This implies that the relatively high PSII activity under anaerobic conditions is an important reason for improved H_2_ photoproduction in the stepwise mode.

### The Stepwise Mode Slightly Enhances the Activities of Two Alternative Electron Sinks for H_2_ Photoproduction

**FIGURE 6:**
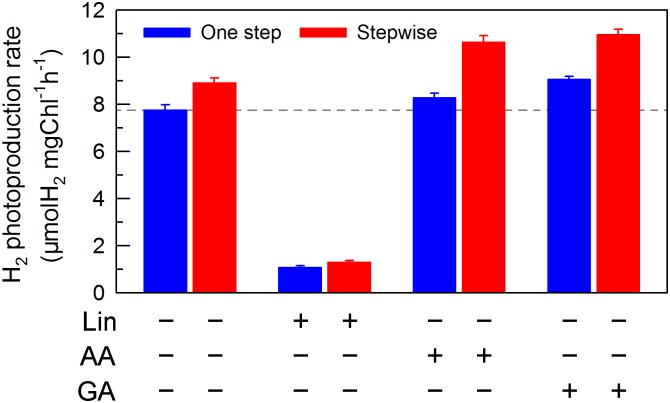
Comparison of the H_2_ photoproduction in *C. reinhardtii* between one step method and stepwise mode with multiple inhibitors. After cells were statically pre-cultured under continuous illumination of 200 μmol photons m^-2^s^-1^ for 36 h, NaHSO_3_ was added as described in the legend for Figure 1, as well as several inhibitors, lincomycin (Lin; 5 mM), antimycin A (AA; 10 μM), and glycolaldehyde (GA; 2 mM) were also added to the serum bottles, respectively. Values are means ± SD (*n* = 5).

We also measured the activities of CET and CO_2_ assimilation, two alternative electron sinks for H_2_ photoproduction. The rates of CET and CO_2_ assimilation were slightly faster in the stepwise mode than those in the one step method, as estimated by the rate of re-reduction of P700^+^ (Figure 4), and photosynthetic production of O_2_ with NaHCO_3_ as an artificial electron acceptor (Figure 5), respectively. It appears plausible that at least the two alternative electron sinks for H_2_ photoproduction do not contribute to enhance the photoproduction of H_2_ in the stepwise mode. If this possibility is true, an increase in H_2_ photoproduction caused by impaired the activity of either CET or CO_2_ assimilation will be higher in the stepwise mode than that in the one step method. The results shown in Figure 6 support our hypothesis that the increase in H_2_ photoproduction was slightly higher in the stepwise mode than that in the one step method in the presence of either AA that specifically inhibits the CET activity (Tagawa et al., 1963) or GA that disrupts the Calvin–Benson cycle activity via inhibiting the phosphoribulokinase (Rühle et al., 2008). Taking all these results together, we may conclude that in the anaerobic background, increased residual PSII activity can significantly enhance the yield of H_2_ photoproduction in *C. reinhardtii*.

If this conclusion is true, impaired PSII activity in an anaerobic environment created by NaHSO_3_ addition will inevitably decrease the yield of H_2_ photoproduction in *C. reinhardtii* at a significant level. As expected, the H_2_ photoproduction rate was significantly decreased in the presence of Lin, which impairs the PSII activity via inhibiting the D1 protein synthesis (Vavilin et al., 1995), regardless of either one step method or stepwise mode (Figure 6). This consolidates our conclusion that increased residual PSII activity in an anaerobic environment is an efficient strategy to improve H_2_ photoproduction in *C. reinhardtii* and the stepwise NaHSO_3_ addition mode is a case study in this strategy.

## Discussion

Whether NaHSO_3_ addition promotes photosynthesis or H_2_ photoproduction depends on its concentrations: NaHSO_3_ in a low amount improves photosynthesis (Wang et al., 2003) but in a moderate amount can enhance H_2_ photoproduction (Wang et al., 2010; Ma et al., 2011). Wang et al. (2003) demonstrate that a low amount (100 μM) of NaHSO_3_ increases cyclic photophosphorylation and consequently improves photosynthesis via optimizing the ATP/NADPH ratio. By contrast, Wei et al. (2017) demonstrate that a moderate amount (13 mM) of NaHSO_3_ can remove O_2_ efficiently through the reaction of bisulfite with superoxide anion produced at the acceptor side of PSI, especially under sufficient light conditions, consequently activates H_2_ase and promotes H_2_ photoproduction. The results of this study indicate that a moderate amount of NaHSO_3_ under a stepwise addition mode can quickly establish an anaerobic environment (Figure 2) and significantly improves H_2_ photoproduction in a unicellular green alga *C. reinhardtii* (Table 1 and Figure 1). Such improvement is at least independent of two alternative electron sinks for H_2_ photoproduction, CET (Figures 4, 6), and CO_2_ assimilation (Figures 5, 6) and, most likely the result of maintained a relatively high electron source for H_2_ photoproduction, PSII activity (Figures 3, 6).

Under a photon flux density of 200 μmol photons m^-2^s^-1^, the Mehler reaction is usually considered to also be an important alternative electron sink for H_2_ photoproduction. However, we found that the Mehler reaction is almost absent in NaHSO_3_ addition strategy, regardless of either one step method or stepwise mode (data not shown), possibly because addition of NaHSO_3_ to the cell suspension cultures quickly results in entering of cells to an anaerobic environment (less than 800 s) (Figure 2). Therefore, the improvement of H_2_ photoproduction in *C. reinhardtii* by a moderate amount of NaHSO_3_ under a stepwise addition mode is also independent of a third alternative electron sink for H_2_ photoproduction, Mehler reaction, and consolidating the above mentioned possibility that such improvement is the result of increased residual PSII activity, an electron source for H_2_ photoproduction.

Based on different sources of electrons to H_2_ase, three pathways for H_2_ production have been identified in *C. reinhardtii*. Their sources of electrons to H_2_ase come from water photolysis via PSII (Melis et al., 2000; Kosourov et al., 2003), NADPH through type II NAD(P)H dehydrogenase (Baltz et al., 2014) and the fermentative degradation of endogenous compounds (Gfeller and Gibbs, 1985), respectively. We observed that production of H_2_ under photon flux densities of 200 μmol photons m^-2^s^-1^ by NaHSO_3_ addition was almost completely suppressed in cells incubated in the dark (Supplementary Figures S1A,B) or treated with DCMU (Supplementary Figures S1A,C). A quick establishment of anaerobic environment by NaHSO_3_ addition (Figure 2) suppresses the acetate uptake (Jurado-Oller et al., 2015) and impairs the mitochondrial respiratory electron transport chain function. Taking all these results together, we propose that in the NaHSO_3_ addition strategy, the source of electrons for H_2_ production predominantly, if not totally, comes from water photolysis via PSII, regardless of either the one step method or the stepwise mode.

The results of this study further indicate that the stepwise mode increased the maximum accumulated H_2_ levels, produced a higher maximum velocity of H_2_ photoproduction, and prolonged the time length of H_2_ photoproduction when compared to the one step method (Table 2). We thus propose that the stepwise mode developed in this study is an efficient and sustained strategy for improving H_2_ photoproduction in the green alga *C. reinhardtii*.

Although a moderate amount of NaHSO_3_ can remove efficiently O_2_, the activity of PSII, an electron source for H_2_ photoproduction, is also significantly impaired (Wang et al., 2010; Ma et al., 2011; Wei et al., 2017). The results of this study observe that in the anaerobic background, a stepwise mode maintains a relatively high PSII activity (Figure 3) and consequently promotes H_2_ photoproduction (Figure 1). The cause and effect of PSII activity and H_2_ photoproduction is also present in the sulfur-deprived strategy (Zhang et al., 2002; Kosourov et al., 2005; Kim et al., 2010; Volgusheva et al., 2013; Grewe et al., 2014; Steinbeck et al., 2015; Chen et al., 2016) but the reasons why H_2_ photoproduction is terminated in sulfur deprivation and NaHSO_3_ addition strategies are distinctly different. It is known that H_2_ photoproduction is terminated in the sulfur deprivation strategy because of cell death (Nguyen et al., 2008) and in the NaHSO_3_ addition strategy because of conversion of too much bisulfite to sulfate (Wei et al., 2017). It is worthy of note that the relationship between H_2_ production and biomass accumulation in sulfur deprivation and NaHSO_3_ addition strategies is also distinctly different. Regardless of either one step method or stepwise mode, the simultaneous production of H_2_ and biomass is present in NaHSO_3_ addition strategy, as observed in mixotrophic nutrient-replete cultures under low light conditions (Jurado-Oller et al., 2015), but is absent in sulfur deprivation strategy. Therefore, it appears reasonable that improved PSII activity in the NaHSO_3_ background is considered to be a better strategy to meet future application requirements in comparison with that in the sulfur-deprived background.

## Author Contributions

WM designed and supervised the experiments. XL, BF, and ZR performed the experiments and analyzed the data. LW and WM analyzed and interpreted the data and wrote the article.

## Conflict of Interest Statement

The authors declare that the research was conducted in the absence of any commercial or financial relationships that could be construed as a potential conflict of interest.

## Abbreviations

AA: antimycin A
CET: cyclic electron transport around PSI
Chl: chlorophyll
*C. reinhardtii*: *Chlamydomonas reinhardtii*
DCMU: 3-(3,4-dichlorophenyl)-1,1-dimethylurea
DO: dissolved oxygen
*F*_v_/*F*_m_: maximal quantum yield of PSII
GA: glycolaldehyde
H_2_ase: hydrogenase
Lin: lincomycin
MV: methyl viologen
one step method: a one step addition method of NaHSO_3_
stepwise mode: a stepwise addition mode of NaHSO_3_
TAP: Tris-acetate-phosphate
