# Supplementary material for: A Stepwise NaHSO3 Addition Mode Greatly Improves H2 Photoproduction in Chlamydomonas reinhardtii
Source: Front Plant Sci. 2018 Oct 31;9:1532. doi: 10.3389/fpls.2018.01532 (PMC6220153; doi:10.3389/fpls.2018.01532)
Supplement: Supplementary file 1 [file Image_1.pdf]

SUPPLEMENTARY MATERIAL

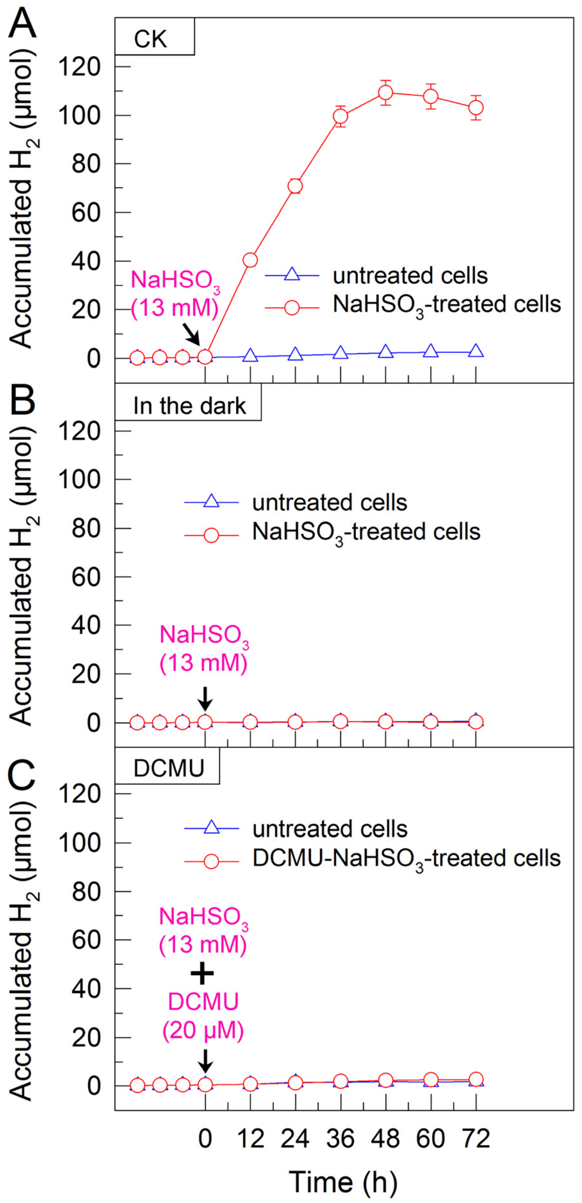

Supplementary Figure S1 Effects of dark incubation and DCMU addition on H_2 production in $NaHSO_3$-treated cells of *C. reinhardtii*. Production of H_2 under photon flux densities of 200 μmol photons $m^{-2}s^{-1}$ by $NaHSO_3$ addition **(A)** was almost completely suppressed in cells incubated in the dark **(B)** or treated with DCMU **(C)**. Values are means ± SD (n = 6). CK, control check.
